# Development and Characterization of *In Situ* Oral Gel of Spiramycin

**DOI:** 10.1155/2014/876182

**Published:** 2014-06-24

**Authors:** Avinash Sharma, Jyoti Sharma, Rupinder Kaur, Vinay Saini

**Affiliations:** Department of Pharmaceutics, S. D. College of Pharmacy, Barnala, Punjab 148101, India

## Abstract

The present investigation deals with the optimization, formulation, and characterization of oral *in situ* gel of spiramycin. Sodium alginate and hydroxypropyl methylcellulose were used as cross-linking and viscosifying agents, respectively. Sodium bicarbonate was used as a floating agent. In preformulation studies, the melting point, pH, and partition coefficient were found to be 133°C, 9.5, and 0.193, respectively. The drug had retention time at around 2.65 minutes in high performance liquid chromatography (HPLC). During compatibility studies of drug with all polymers, we observed that there were no changes in the FTIR spectra of a mixture of drug and polymers. All the formulations showed good pourability. Floating time and total floating time were ~30 sec and >12 hours, respectively. During *in vitro* drug release studies, the drug was released from the formulation around 80–100% for 12–16 hrs. In TEM analysis, we found that the drug molecules were well entrapped in the polymer and the drug was released slowly for up to 12 hrs. In these studies, we found that the concentration of sodium alginate and HPMC had significant influence on floating lag time, gelling capacity, and cumulative percentage drug release. During antimicrobial studies, we found that the formulation containing spiramycin showed good zone of inhibition against different microbial strains (*Staphylococcus aureus* and *Escherichia coli*).

## 1. Introduction

Antimicrobial therapy of an infection ultimately depends on the concentration of the antibiotic at the site of infection, which must be sufficient to inhibit growth of the offending microorganisms. If host defenses are intact, agents that interfere with growth or replication of the microorganism but do not kill it (i.e.,* bacteriostatic* agents) may suffice [1–3].* Staphylococcus aureus* is a bacterium that is a member of the Firmicutes and is frequently found in the human respiratory tract and on the skin [1, 4]. Although* Staphylococcus aureus* is not always pathogenic, it is a common cause of skin infections (e.g., boils), respiratory disease (e.g., sinusitis), and food poisoning.* Staphylococcus aureus* food poisoning is often caused when a food handler contaminates food products that are served or stored at room temperature or refrigerator temperature [1, 2]. Sign and symptoms usually appear within 1–6 hours after eating contaminated food. Other infections caused by* Staphylococcus aureus* are skin and soft tissue infections such as abscesses, cellulitis, and pneumonia [3].* Staphylococcus aureus* infection is diagnosed by culture of infected material using gram staining. Initial choice and dosage of antibiotics depend on infection site, illness severity, and probability that resistant strains are involved. Spiramycin is a white or slightly yellowish powder with slight odour and slightly hygroscopic macrolide antibiotic. It is used to treat toxoplasmosis and various other infections of soft tissues [3, 5]. Spiramycin may be taken with food if stomach upset occurs. For best results, take each dose at evenly spaced intervals. This will ensure a constant level of medication in your blood. Take this medication for the full time prescribed. Do not stop taking this without your doctor's approval. Stopping therapy too soon may result in reinfection [5] and side effects that include indigestion, nausea, vomiting, diarrhea, or stomach ache may occur. The use of this medication for prolonged or repeated periods may result in a secondary infection (e.g., oral, bladder, or vaginal yeast infection) [5, 6]. This drug should be used only if clearly needed during pregnancy. Small amounts of drug do appear in breast milk, so consult your doctor before breast-feeding [6]. Controlled release dosage forms cover a wide range of prolonged action formulations which provide continuous release of their active ingredients at a predetermined rate and for a predetermined time [7]. A variety of controlled release drug products have been designed with specific therapeutic objectives based on the physiochemical, pharmacological, and pharmacokinetics properties of the drug [8]. Gastroretentive system ensures that whole drug delivery system remains within the gastric region for longer duration of time [9]. Floating drug delivery systems meant for gastric retention float on the surface of the gastric fluids, due to their low density, and produce prolonged effect by showing the release, while being buoyant on gastric fluid surface. This type of delivery system is of great value for drugs which get absorbed from upper part of the stomach; that is, their absorption window resides in upper part of stomach [9, 10]. In these studies, we describe the formulation and evaluation of* in situ* oral gels of spiramycin based on the concept of pH triggered and ion activated systems. The system utilizes polymers that exhibit sol-to-gel phase transition due to change in specific physicochemical parameters. A pH triggered* in situ* gel consisting of sodium alginate (cross-linking agent), sodium bicarbonate(floating agent) bearing unique properties of hydroxypropyl methylcellulose (HPMC) to prolonging the release of spiramycin from* in situ* gel.

## 2. Materials and Methods

### 2.1. Materials

Spiramycin was a gift sample from Corona Remedies Pvt. Ltd. And hydroxypropyl methylcellulose was purchased from Central Drug House (P) Ltd., New Delhi. Sodium alginate and calcium chloride were purchased from Qualikems Fine Chemicals Pvt. Ltd., New Delhi (India). Sodium bicarbonate and sodium citrate were purchased from Nice Chemicals Pvt. Ltd., Cochin (India). All other materials and chemicals used were of either pharmaceutical or analytical grade.

### 2.2. Microbial Culture

*S. aureus and E.coli* were used in culture.

### 2.3. Methods

#### 2.3.1. Synthesis of* In Situ* Oral Gel (Preparation of* In Situ* Gelling Solution)

Sodium alginate, at different concentrations (2% w/v, 2.5% w/v, and 3% w/v), was prepared in deionised water containing calcium chloride (0.15% w/v) and sodium citrate (0.5% w/v). After that, hydroxypropyl methylcellulose K100 (0.3% w/v, 0.6% w/v, and 0.9% w/v) was added to it. The sodium alginate solution was heated to 50°C with stirring. After cooling (<40°C), Calcium carbonate (1.5% w/v) and spiramycin were added and dispersed well with continuous stirring (see Table 1). The resulting* in situ* gel solution containing spiramycin was finally stored in amber color narrow mouth bottles until further use [11].

*Preformulation Study of Spiramycin and Compatibility Studies of Spiramycin with Polymer.* Preformulation studies (the melting point, solubility, pH, partition coefficient of drug, and HPLC) and compatibility studies (using Fourier transform infrared spectroscopy (FTIR)) of spiramycin with polymers were performed prior to the preparation of* in situ* gel. The individual FTIR spectrum of the pure drug and combined spectra of the drug with sodium alginate and HPMC were also performed.

*Characterization of In Situ Oral Gel*

*(i) Appearance.* All formulations were evaluated for clarity by visual observation against a black and white background [11].

*(ii) pH.* The pH values of different formulations were measured using a calibrated digital pH meter at room temperature in triplicate [12].

*(iii) In Vitro Gelation Studies.* Gelation of* in situ* gelling solution was carried out by taking 500 mL of 0.1 N hydrochloric acid (HCL; pH 1.2) in a beaker. 10 mL of solution was added to HCL with mild agitation to avoid breaking of formed gel. Gelling was observed visually by qualitative measurement [11].

*(iv) Measurement of Viscosity of In Situ Gelling Solution.* The viscosities of the prepared gel formulations (F-9 to F-13) were determined by digital brook field viscometer. The samples (100 mL) were sheared at a rate of 100 rpm using suitable spindle at room temperature. Viscosity measurement for each sample was done in triplicate, while each measurement was taken approximately for 30 seconds [11].

*(v) Determination of Drug Content.* The drug content in each unit dosage form was determined by UV spectroscopy. The UV absorbance of the sample was determined at a wavelength of 231 nm. The drug content for batches was measured in triplicate and the average values were recorded [13].

*(vi) In Vitro Floating Studies.* Floating studies of* in situ* gelling solution were carried out in 500 mL of 0.1 N HCL (pH 1.2) in a beaker. 10 mL of solution was added to HCL with mild agitation. Time required for floating on the surface after adding solution (floating lag time) and total floating time were measured [11].

*(vii) In Vitro Drug Release Studies.* The* in vitro* release rate of spiramycin from sustained release* in situ* gel was performed in USP apparatus fitted with paddle (50 rpm) at 37 ± 0.5°C. 0.1 N HCL (500 mL) was used as a dissolution medium. This speed was kept slow enough to avoid the breaking of gelled formulation under mild agitation conditions similar to physiological salt conditions. 10 mL sample was withdrawn and filtered through a 0.45*μ*m membrane filter. At the predetermined time intervals the samples were assayed at 232 nm using a Shimadzu UV-1800 double-beam spectrophotometer. Percentage cumulative drug release (% CDR) was calculated using an equation obtained from a calibration curve [11].

*(viii) Transmission Electron Microscopy.* Morphology of spiramycin loaded* in situ* gel was observed under transmission electron microscopy (TEM). One drop of diluted* in situ* suspension containing spiramycin was put on a film-coated copper grid and stained with one drop of 2% (w/v) aqueous solution of phosphotungstic acid. The sample was allowed to dry for contrast enhancement. The sample was then examined by transmission electron microscopy. 

*(ix) Stability Studies.* Prepared solutions were first packed in glass bottles and kept for three months. The stability of the* in situ* gels was monitored for 3 months at accelerated stability. Periodically (initially for 1.0, 2.0, and 3.0 months intervals) samples were removed and characterized by physical appearance, pH, and drug content [14]. 

*(x) Zone of Inhibition Test for Antimicrobial Activity.* The antimicrobial studies were carried out for the plain drug (control) as well as for different formulations (having spiramycin 50, 25, 12.5, and 6.25 mg/mL). These studies were performed using two different test microorganisms such as* Staphylococcus aureus* and* Escherichia coli* for antimicrobiological studies. The nutrient agar (20 mL) was seeded as a layer with the test microorganism (0.2 mL) which was allowed to solidify in the Petri plate. Cups were made on the solidified agar layer with the help of sterile borer at 6.0 mm diameter. Appropriate amount of drug loaded gel solution was poured into the cups. Petri plate was kept at room temperature for 4 hours and the plates were incubated at 37°C for 24 hours. The zone of inhibition was measured. The diameter of zone (mm) of inhibition was measured by an antibiotic zone finder [14].

## 3. Results and Discussion

**Figure 1:**
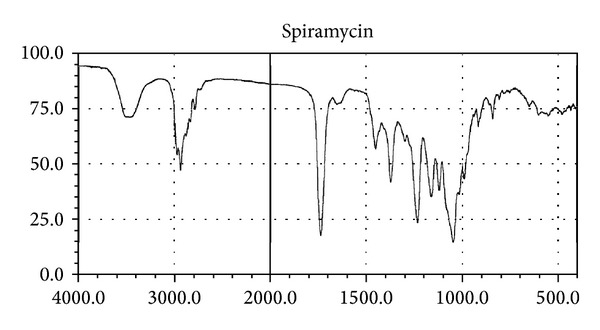
Fourier transform infrared spectrum of pure spiramycin.

In preformulation studies, the melting point, pH, and partition coefficient were found to be 133°C, 9.5, and 0.193, respectively. FTIR spectrum of the pure spiramycin was recorded by FTIR spectrophotometer. Results are shown in Figure 1.

**Figure 2:**
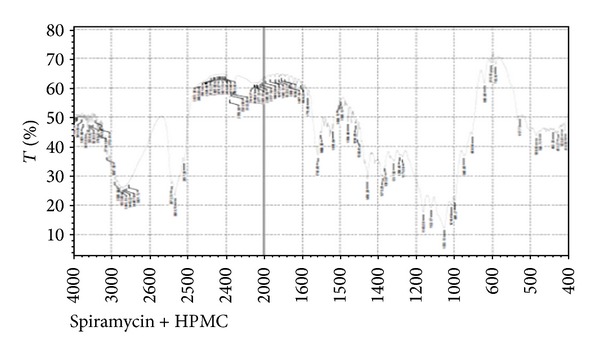
Fourier transform infrared spectra of spiramycin with HPMC.

**Figure 3:**
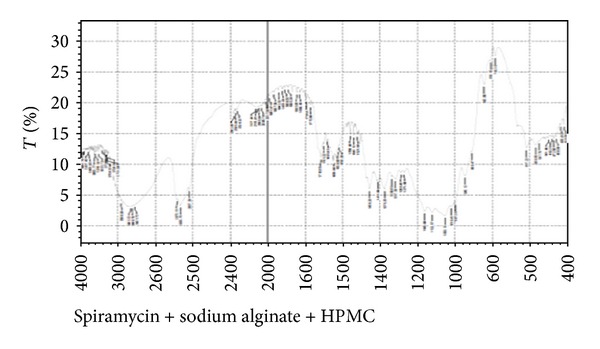
Fourier transform infrared spectra of spiramycin with HPMC and sodium alginate.

In compatibility studies of drug with polymers, the drug shows bands for its different groups, namely, ester (1275 cm^−1^), alcoholic (1090 cm^−1^), aldehydic (1735 cm^−1^), dimethyl amino, and hydroxyl (broad) group. The peaks were well separated from the peaks formed for polymers. Combined FTIR spectra of drug with polymers are shown in Figures 2 and 3.

**Figure 4:**
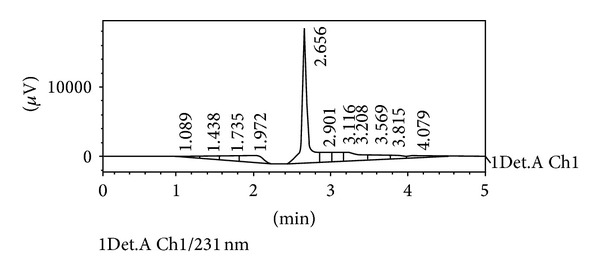
HPLC chromatogram of spiramycin.

*High Performance Liquid Chromatography of Drug.* HPLC of spiramycin was performed. The retention time of spiramycin was found at around 2.656 min. Data are shown in Figure 4.

**Figure 5:**
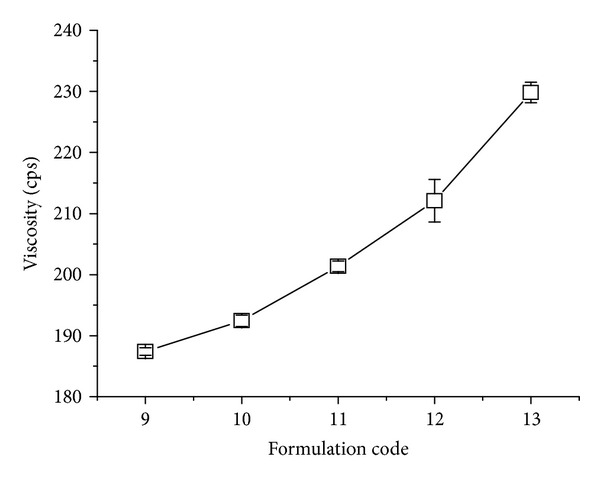
Viscosity of different formulations (F-9 to F-13).

*pH, Drug Content, In Vitro Gelation, Floating Studies, and Viscosities of In Situ Gel.* Data are recorded in Table 3 and shown in Figure 5.

**Figure 6:**
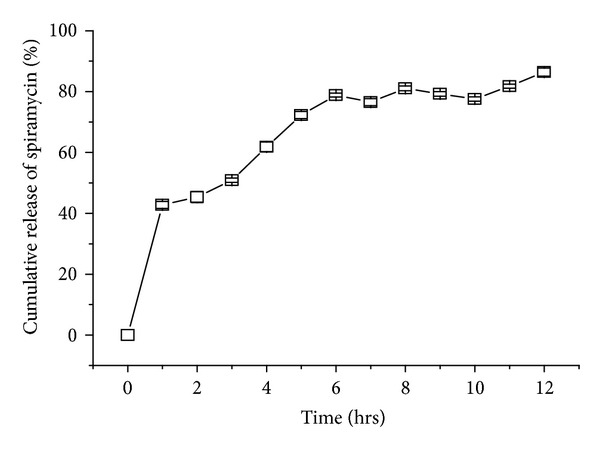
Percentage cumulative release of spiramycin from hydrogel (F-12).

*The Percentage Cumulative Release of Drug from In Situ Gel.* At different time intervals (from 0 to 12 hrs), the percentage cumulative release of drug from best formulation (F-12) is shown in Figure 6. 

**Figure 7:**
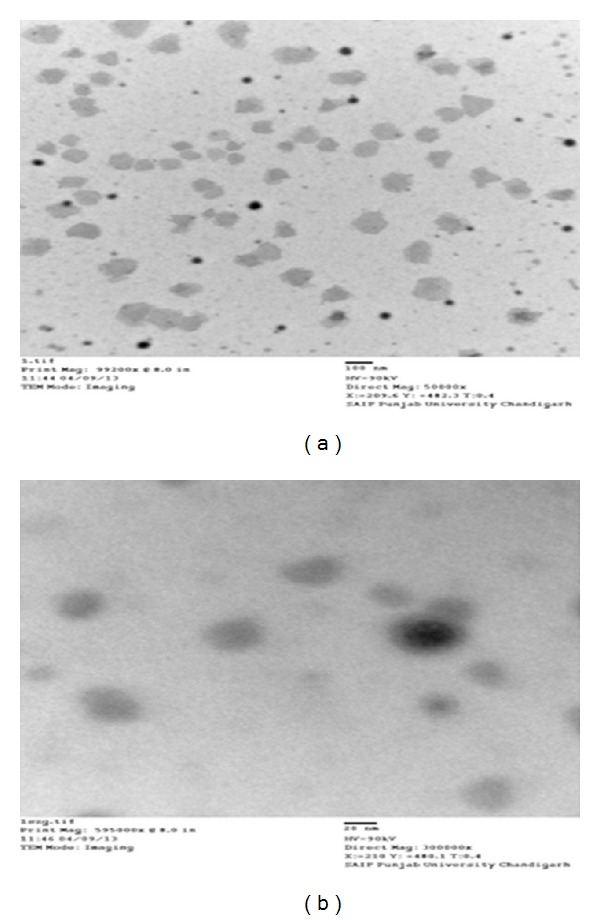
Transmission electron microscopy of spiramycin loaded formulation (F-12).

*Transmission Electron Microscopy.* TEM photographs show distribution of drug inside* in situ* gel. Results are shown in Figure 7.

*Stability Studies of In Situ Formulation.* Effects on pH and drug content (%) on storage at different time periods are recorded in Table 4.

**Figure 8:**
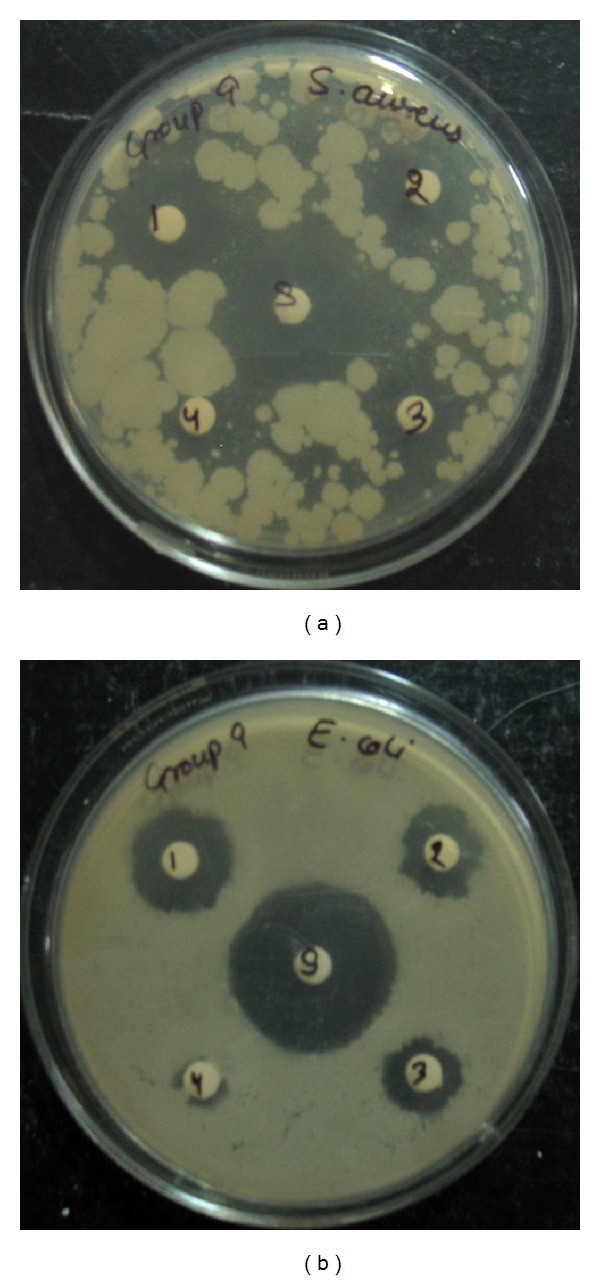
Different concentrations of* spiramycin loaded in situ gels incubated with Staphylococcus aureus and E. coli, respectively* (where dilutions are referred to as 50 mg/mL, 2.25 mg/mL, 6.25 mg/mL, and 5.0 mg/mL).

**Figure 9:**
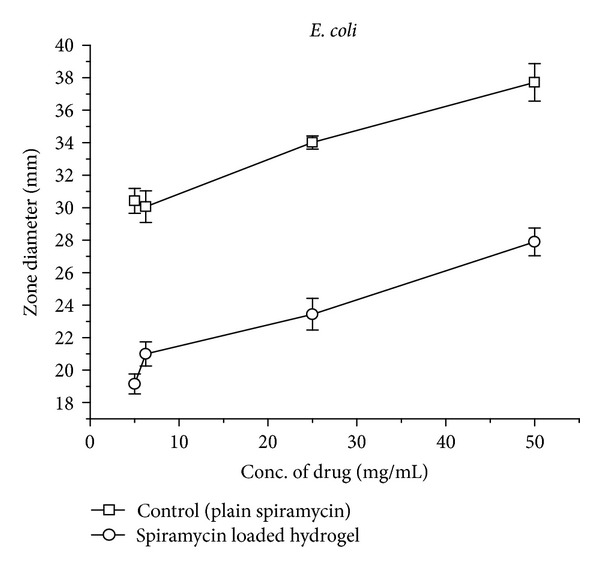
Graph showing comparison of antimicrobiological activities (zone diameter) for plain spiramycin and spiramycin loaded hydrogel (F-12) against* E. coli.*

**Figure 10:**
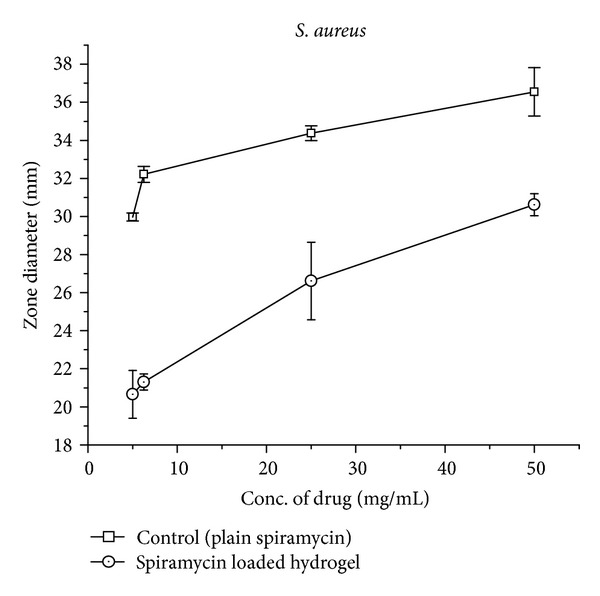
Graph showing comparison of antimicrobiological studies (zone diameter) of plain spiramycin and spiramycin loaded hydrogel (F-12) against* Staphylococcus aureus* (B).

*Antimicrobial Studies.* The assay was used to test the ability of spiramycin (antibiotic) and antibiotic loaded gel formulations in the microbial growth. The zone of inhibition test method was designed to qualitatively test the ability of drug and spiramycin loaded gel formulations to inhibit the microbial growth. The assay was performed for around 18–24 hours. Results are shown in Figure 8 and recorded in Figures 9 and 10.

## 4. Discussion

Spiramycin belongs to the class of antibiotics used in the treatment of respiratory tract infections, stomach infection, and so forth. Generally the high dose of spiramycin is difficult to incorporate in floating tablets, capsules, and so forth which can be easily given in liquid or semisolid dosage form, namely,* in situ* gel. The pH of the formulations was found in the range of 7.51–7.58. Spiramycin is freely soluble in certain solvents, namely, water, acetone, and methanol. Melting point of spiramycin was around 133°C indicating the purity of the drug sample [7]. The compatibility studies of drug with polymers were determined by comparing the FTIR spectra of plain spiramycin and spiramycin with different polymers which were used in the formulations [15]. The drug shows its peak due to the presence of ester (1275 cm^−1^), alcoholic (1090 cm^−1^), aldehydic (1735 cm^−1^), dimethyl amino, and hydroxyl (broad) group. It was observed that there were no changes in these main peaks in the FTIR spectra of drug and polymers. It can be concluded that the drug maintains its identity without any chemical interaction with the polymers used. The blank* in situ* gels (F-1–F-16) were also formulated using different concentrations of sodium alginate and hydroxypropyl methylcellulose (see Table 2). Sodium alginate was used at 2.5% w/v and it shows good gelation (+++) time, floating lag time of 13–20 seconds, and also duration of floating time of 10–12 hours. This might be due to the reaction of sodium alginate with sodium citrate to form the good gelling properties. Therefore, sodium alginate was selected as the best* in situ* gelling polymer for further investigations [15].* In vitro* drug release profile studies were performed in our best formulation (F-12). It showed that around ~85% of drug was released for 12 hrs in a controlled manner as drug molecules were entrapped in the polymers and cross-linked into gel. This might be due to HPMC which increases the viscosity of the formulation and therefore modifies the release of drug from gel. Different formulations (F-9 to F-16) show good gelling properties as they maintain the concentration of sodium alginate (2.5% w/v) along with sodium citrate. Sodium bicarbonate exhibited good floating lag/total time due to good effervescent properties [11]. The % drug content in different formulations was found to be in the range of 83–87 [13]. Among all the formulations, formulations F-12 to F-14 showed excellent percentage drug release, gelling capacity, and floating time as compared to other formulations. When the concentration of sodium alginate and HPMC was kept low the viscosity, the gelling capacity of the formulations was found to be decreased. On increase in the concentration of sodium alginate (3% w/v) and HPMC (0.9% w/v), the percentage drug release was also increased with low gelling properties because, on increasing the concentration of sodium alginate and HPMC, the viscosity of formulations is also increased while gelling capacity of the formulation is decreased. Formulations F-12 to F-14 showed good percentage drug release and gelling capacity because the concentration of sodium alginate and HPMC was kept optimum for percentage drug release as well as for gelling properties of the formulation [15]. The stability studies of best optimized formulation (F-12) were carried out for three months. In these studies, drug content, pH of the formulation was evaluated and formulation found to be stable also in physical appearance when stored for 3 months [13, 15]. In TEM analysis, we found that the drug molecules were entrapped in the polymers and cross-linked into gel. The microbiological studies show that the formulations showed good zone of inhibition against microbial strains of* Staphylococcus aureus* and* Escherichia coli*. In these studies, we found that the diameter of zone of inhibition increased with concentration of drug [14]. Ciprofloxacin was used as a positive standard (10 microgram/mL) and was found to be better for* E*.* coli* than for* S. aureus*. In these studies, we have noticed that there was quite difference in the antimicrobial activities of plain spiramycin as compared to drug loaded gel. However, these studies were performed in* Staphylococcus aureus* and* Escherichia coli,* but the research work was envisaged to develop spiramycin loaded* in situ* oral gel against toxoplasmosis which is a fatal protozoan disease but due to the unavailability of the strain of toxoplasma* gondii* in India and, thus, we performed these studies in different microbial strains. In near future,* in situ* oral gel should be applied to enhance the potency of antibiotics against* Toxoplasma gondii* (toxoplasmosis). After performing these studies, it can be concluded that the good application of* in situ* oral gel is to administer antibiotics where the system will simulate with acidic pH of the stomach. The sol-gel system will be helpful in prolonging its floating time and, thus, retention time in acidic environment where it will release the drug in a controlled manner for long time without compromising the stability of orally administered antibiotics. Finally, the* in situ* oral gel may be helpful in increasing the potency of orally administered antibiotics.

## Figures and Tables

**Table 1:** Preparation of spiramycin *in situ *oral gel using different concentrations of calcium chloride, sodium citrate, and sodium bicarbonate.

Quantity in 100 mL (concentration in % w/v)									
S. number	Ingredients	F-1	F-2	F-3	F-4	F-5	F-6	F-7	F-8
1	Drug	5.00	5.00	5.00	5.00	5.00	5.00	5.00	5.00
2	Calcium chloride	0.050	0.075	0.10	0.15	0.15	0.15	0.15	0.15
3	Sodium citrate	0.25	0.30	0.35	0.40	0.45	0.5	0.5	0.5
4	Sodium alginate	2.5	2.5	2.5	2.5	2.5	2.5	2.5	2.5
5	HPMC	0.8	0.8	0.8	0.8	0.8	0.8	0.8	0.8
6	Sodium bicarbonate	1.5	1.5	1.5	1.5	1.5	0.5	1.0	2.0

**Table 2:** Preparation of spiramycin *in situ *oral gel using different concentrations of sodium alginate and HPMC.

Quantity in 100 mL (concentration in % w/v)									
S. number	Ingredient	F-9	F-10	F-11	F-12	F-13	F-14	F-15	F-16
1	Drug	5.00	5.00	5.00	5.00	5.00	5.00	5.00	5.00
2	Calcium chloride	0.15	0.15	0.15	0.15	0.15	0.15	0.15	0.15
3	Sodium citrate	0.5	0.5	0.5	0.5	0.5	0.5	0.5	0.5
4	Sodium alginate	2.0	2.0	2.0	2.5	2.5	2.5	3.0	3.0
5	HPMC	0.3	0.3	0.3	0.6	0.6	0.6	0.9	0.9
6	Sodium bicarbonate	1.5	1.5	1.5	1.5	1.5	1.5	1.5	1.5

**Table 3:** pH, drug content, *in vitro* gelation, and floating studies of different formulations.

Formulations	pH	Drug content (percentage)	Gelling studies	Floating lag time (seconds)	Duration of floating (hours)
F-9	7.54 ± 0.010	84 ± 0.26	++	18.0	>12
F-10	7.56 ± 0.004	87 ± 0.22	++	15.0	>12
F-11	7.55 ± 0.010	83 ± 0.14	++	13.0	>12
F-12	7.53 ± 0.002	86 ± 0.20	+++	21.0	>12
F-13	7.58 ± 0.12	84 ± 0.12	+++	15.0	>12
F-14	7.51 ± 0.002	83 ± 0.20	+++	10.0	>12
F-15	7.56 ± 0.008	85 ± 0.14	++	12.0	>12
F-16	7.54 ± 0.006	87 ± 0.10	++	16.0	>12

(++) Gelation immediate remains for 12 hours.

(+++) Gelation immediate remains for more than 12 hours.

**Table 4:** Stability studies of *in situ *gelformulation (F-12).

Time period for sampling	pH	Drug content (%)
Initial	7.53 ± 0.120	86 ± 0.12
After 1 month	7.51 ± 0.110	85 ± 0.14
After 2 months	7.53 ± 0.005	84 ± 0.20
After 3 months	7.50 ± 0.105	82 ± 0.10
